# Issues that cannot be ignored: the decline in adropin over the aging process

**DOI:** 10.1590/1806-9282.20241186

**Published:** 2025-03-17

**Authors:** Yanting Zhang, Penghao Yang

**Affiliations:** 1 Yiwu Central Hospital, Operating Room – Yiwu, China.; 2 Yiwu Central Hospital, Department of Emergency – Yiwu, China.

Dear Editor,

We are pleased with the study by Tataroğlu et al.^1^, which showed that elevated levels of adropin in trauma patients suggest that it triggers protective mechanisms. But I think some issues still need to be addressed.

The main problem with this study is that the confounding factor of age was not taken into account. Patients were divided into two groups based on adropin levels. Once grouped, there may be age differences between the two groups. Previous studies have shown that serum adropin levels decrease with age^2^. Adropin, a peptide discovered in 2008, has gained recognition as a key regulator of cardiovascular health and metabolic balance^3^. The study also found that patients with normal blood pressure had higher levels of adropin than other patients. This is consistent with previous studies showing that reduced plasma norepinephrine levels are associated with increased blood pressure in hypertensive patients^4^. It is well known that age is also an independent risk factor for hypertension. Therefore, age should be considered a confounding factor when analyzing the association of adropin with patients with traumatic brain injury and hypertension.

In summary, there are many factors (such as age) that influence serum adropin levels. Age must be excluded as a confounding factor when examining the association between serum adropin and patients with traumatic brain injury.
